# Addressing the implementation gap in advanced therapeutics for spinal muscular atrophy in the era of newborn screening programs

**DOI:** 10.3389/fneur.2022.1064194

**Published:** 2022-12-12

**Authors:** Carmen Leon-Astudillo, Barry J. Byrne, Ramzi G. Salloum

**Affiliations:** ^1^Department of Pediatrics, University of Florida College of Medicine, Gainesville, FL, United States; ^2^Department of Health Outcomes and Biomedical Informatics, University of Florida College of Medicine, Gainesville, FL, United States

**Keywords:** spinal muscular atrophy (SMA), gene therapy, implementation science, nusinersen, risdiplam, onasemnogene-abeparvovec, newborn screening program

## Abstract

Spinal muscular atrophy (SMA) is a rare genetic disease that results in progressive neuromuscular weakness. Without therapy, the most common form of the disease, type 1, typically results in death or chronic respiratory failure in the first 2 years of life. Thanks to the recent introduction of newborn screening programs and the discovery of three disease-modifying therapies in the last decade, the outcomes of children with SMA have dramatically improved. Patients are able to achieve many, if not all, of the typical neuromotor milestones, such as sitting, standing and walking, as well as safe oral intake. As the natural history of treated patients is continuously evolving, children with SMA continue to require complex and multidisciplinary care, posing implementation and sustainability challenges. Accordingly, there is a significant need for the application and evaluation of implementation science to address the steps involved in the diagnosis and treatment of patients with SMA, ensuring that all pertinent stakeholders and systems are working effectively to deliver timely and appropriate care. In this manuscript, we discuss the current challenges and gaps in the care for children with SMA, as well as how implementation science can advance this field. In addition, we provide an adapted implementation science framework that includes the main domains and subdomains involved in the care of patients with SMA.

## Introduction

Spinal muscular atrophy (SMA) is an autosomal recessive disease with an incidence of approximately 1 in 10,000 live births (1). SMA is caused by biallelic mutations in the Survival Motor Neuron 1 (SMN1) gene resulting in early onset degeneration of α-motor neurons in the anterior horn of the spinal cord with secondary progressive muscle weakness and atrophy (1). Thanks to advances in newborn screening programs, SMA can now be detected in the first week of life. Despite a lack of evidence-based therapies for SMA, the era of precision medicine holds promise for the implementation of advanced therapeutics to treat SMA. If implemented well, precision medicine approaches could have a significant benefit to patients with SMA and other rare diseases. However, biomedical science faces a formidable challenge in evaluating the clinical utility of scientific breakthroughs in rare diseases, especially if it follows the traditional path of innovation development, then efficacy and effectiveness studies, prior to implementation (2).

## Background

The best known predictor of SMA severity is the number of copies of Survival Motor Neuron 2 (SMN2), a back-up gene to SMN1, which varies across the population (3). Historically, the disease has been classified into five different types based on achieved neuromotor milestones. *Type 0* presents with very severe hypotonia at birth, has a life expectancy of < 6 months and affected infants usually have 1 SMN2 copy. *Type 1* is clinically diagnosed in the first six months of life and affected patients are unable to sit or stand and typically have 2 or 3 SMN2 copies. *Type 2* has its onset between 6 and 18 months of life, whereby affected children reach the milestone of sitting but do not stand or walk. Patients with type 2 disease have a life expectancy of 10 to 40 years and usually have 3 SMN2 copies. *Type 3* presents after 18 months of life, and children are able to sit, stand and walk with assistance, reach adulthood and tend to have 3 to 4 SMN2 copies. Lastly, *type 4*, the mildest form, is typically diagnosed after the age of 5, whereby patients are able to walk unassisted, are usually diagnosed in adulthood, and carry 4 or more SMN 2 copies (4).

Natural history studies of the most common form, type 1, report a median age at death or the need for non-invasive ventilation of 10.5 months and only 8% of patients survive without respiratory support by 20 months (5). SMA is part of a long list of rare diseases that have benefited from advances in drug development, in part due to the US Orphan Drug Act (1983) that introduced incentives for the pharmaceutical industry to invest in therapies for rare diseases (6, 7). The orphan disease and drug concept follows the principle that people with rare diseases should have equal access to treatment, regardless of the prevalence of the disease. In the specific case of SMA, three therapies have been approved in the past 6 years: nusinersen, onasemnogene-abeparvovec, also known as “gene therapy,” and risdiplam (8–10).

Nusinersen was the first approved drug for SMA. It is an antisense oligonucleotide that binds to the splicing site on the SMN2 pre-mRNA. The modified mRNA is translated into functional SMN protein (10). Onasemnogene-abeparvovec, with the commercial name of Zolgensma^®^ is an adeno-associated viral vector containing the gene that encodes the SMN protein, with an enhancer and promoter for proper gene function. It uses AAV9 or adeno-associated virus 9 to deliver the gene to the affected neurons as a one-time dose. Zolgensma^®^ received orphan drug designation in 2019 (8). The most recent drug approved in 2020, risdiplam, is an mRNA splicing modifier for SMN2 that increases the production of SMN protein (9). Although none of these therapies constitutes a cure, significant improvements in survival, neuromotor function and quality of life are evident (8–10). In addition to these groundbreaking therapies, the recent implementation of newborn screening programs in forty eight states has allowed for early treatment of pre-symptomatic or minimally symptomatic children with SMA (11). However, likely due to the rapid approval of therapies paired with the recent introduction of methods for early diagnosis, a clear gap in implementation has become evident in “real world” settings. Little is known about the long-term implications of these therapies, the ideal healthcare infrastructure required to support their implementation, as well as the medical personnel and resources necessary to provide cost-effective care for these children with complex conditions, given the rapid scientific advancements in the field. Therefore, addressing stakeholder needs, as well as the processes involved in the care of children living with SMA is imperative for optimizing the implementation of SMA interventions and their sustainability.

We herein aim to discuss the main challenges related to the implementation of SMA care, including diagnosis, delivery of therapies, patient outcomes, cost and access, as well as long-term management of SMA.

## From diagnosis to the delivery of therapies

In the era of newborn screening programs, SMA can be diagnosed in the first week of life (12). The program was initially included as part of the recommended uniform screening panel (RUSP) in July of 2018 and has been implemented in all but two states in the United States as of September of 2022, covering approximately 98% of new births (11, 13). In the United States, screening programs are administered at the state level and constitute a complex process that involves multiple organizations and time sensitive algorithms to deliver a timely diagnosis. The Newborn screening process for SMA focuses on the identification of exon 7 deletion in the SMN1 gene, with the goal to identify 95% of newborns with SMA (13). The majority of programs use the multiplex testing that is already utilized for severe combined immunodeficiency testing, a disease that has been part of the RUSP since 2010, which consequently decreases the cost of screening (13).

Environmental factors such as size of the state, population demographics, healthcare infrastructure, practice and provider factors, including laboratory personnel, methodology, medical providers' readiness, preparedness of designated diagnostic centers, as well as the infant and caregivers' characteristics and social determinants can influence the process and should be considered when implementing these interventions.

In some states, such as Florida, only SMN1 copy number is reported by the state laboratory (13). Therefore, early referral is required to assess SMN2 copy number through additional laboratory testing, as well as an urgent clinical encounter with a neuromuscular specialist or team of specialists. At the University of Florida Center for Neuromuscular and Rare Diseases, this step currently includes an appointment with Genetics, Neurology and Pulmonology, along with a video-fluoroscopy study for assessment of swallowing dysfunction and physical therapy evaluation of neuromotor milestones with the use of a validated developmental assessment. During the clinical encounter, treatment options are reviewed with the caregivers and if needed, additional laboratory testing is performed to determine which treatment is indicated. Although there are significant practice variations across the United States, in general, the sense of urgency and need for early evaluation is universal (14).

In addition to age requirements, SMN2 copy number, neuromotor and laboratory testing, insurance approval is a common step and often a significant barrier as approval can often take days to weeks. Some payers can be more restrictive than others by imposing additional criteria to those identified by the Food and Drug Administration (15). Unfortunately, delays in the process can be detrimental to neuromotor function. Once approval is obtained, the treatment center is responsible for the administration of the medication(s). In the case of nusinersen, periodic intrathecal injections performed by a certified physician and laboratory monitoring are required. Zolgensma^®^ administration requires daily oral steroid dosing for roughly 2 months, a specialized pharmacy team, as well as frequent laboratory monitoring after drug administration and titration of steroids based on these and clinical parameters.

Although great progress has been made to date, there continues to be a gap in the implementation of newborn screening for SMA. Screening programs have not reached all states and in addition, there is a lack of clear guidance regarding the minimum or ideal personnel necessary for the adequate execution of this process. For example, specialists need to be involved, and there needs to be an acceptable and ideal time to diagnosis and time to therapy (13, 14).

## Safety of disease modifying therapies

Various studies have reported on the safety of the aforementioned three medications. However, most of the research focuses on their short-term effects. In the case of nusinersen, intrathecal administration carries its own risks and discomforts. Although generally well tolerated, increased intracranial pressure and emesis requiring intubation during anesthesia have been reported (10, 16). Nusinersen is associated with proteinuria, thrombocytopenia and coagulation abnormalities. These laboratory findings tend to be mild and do not change the clinical management of patients living with SMA.

Zolgensma^®^ administration is performed through intravenous infusion, which may require the placement of a central line due to the known abnormal vascularity of affected patients (17, 18). The treatment is associated with transaminitis, steroid responsive hepatotoxicity, complement mediated thrombocytopenia, and thrombotic microangiopathy that recently resulted in a fatality (19–22). An additional problem that has not been uniformly addressed is the variability in the administration of routine vaccinations to infants during steroid administration, with some families choosing to avoid immunizations during steroid treatment, putting the child at risk for fatal, preventable infections.

Risdiplam has been associated with diarrhea, rash, respiratory infections, including pneumonia during clinical trials and animal studies reported potentially reversible fertility problems and retinal toxicity (9, 23, 24). Lastly, although not studied as part of clinical trials, many patients have received two or more therapies with reported higher levels of transaminases in cases that have received Zolgensma^®^ after nusinersen (25).

Long term safety data are scarce given the relative short duration of post marketing approval. Animal studies have raised concern about the risks related to AAV viruses and potential harm related to SMN overexpression at high doses, but literature in humans is lacking (26, 27). Safety monitoring should be the responsibility of the health care teams and pharmaceutical companies, with participation from health insurance companies and governmental agencies, creating a challenging but ideal opportunity for the application of implementation science frameworks.

## Patient outcomes

The efficacy of these medications is evident; however, with three medications on the market, 1:1 comparative effectiveness studies are not feasible and are considered unethical at this point. Although rarely used in rare diseases after drug approval, a Bayesian adaptive trial design could be an option to assess the effect of combination therapies, Althought this design does not fully mitigate the ethical concerns about randomization to the inferior treatment, it reduces the likelihood that patients are allocated to the inferior treatment arm (28). A match-adjusted indirect comparative study of nusinersen vs. Zolgensma^®^ using information from clinical trials (total sample of 82 patients) showed a favorable effect toward Zolgensma^®^, but the small sample and differences across populations limited the generalizability of the results (29). Interestingly, many patients have received two or three therapies as part of their clinical care but it is unclear which patients see more benefit from this approach (30).

It is worth mentioning that although most studies focus on neuromotor milestones, ventilator free time and overall survival, little is known about the efficacy of these therapies in other outcomes such as improvement of dysphagia, degree of sleep disorder breathing and scoliosis (31). Careful use of registries can assist health care professionals and patients when making decisions regarding evaluations and interventions, while optimizing resources.

## Cost and access

Substantial costs are involved in the treatment of SMA, resulting from the newborn screen program implementation to the actual drug dosing, as well as costs associated with short- and long-term monitoring. The novel therapies themselves involve significant costs that are certainly restrictive in many countries. Nusinersen, the first drug approved has a cost of approximately $750,000 during the first year of treatment and $375,000 annually thereafter (32). The cost of a single life-time dose of Zolgensma^®^ is $2.1 million. To date, over 2000 patients have been dosed worldwide (33). Lastly, risdiplam has a weight-based cost that can reach $340,000 annually (34).

Early work has evaluated the cost effectiveness of newborn screening for SMA (35). A study by Shih et al. in Australia demonstrated that newborn screening coupled with gene therapy improved the survival and quality of life for infants with SMA and was cost effective (36). However, given the high price tag and variations in the degree of improvement, particularly in advanced disease, the economic burden of treatment needs to be weighed against expected benefits.

In addition, cost of care beyond therapy administration should be considered, but long-term outcome data are very limited. A literature review estimated the annual cost of care for patients with type 1 SMA, exclusive of drug costs, to range from $75,047 to $196,429 (37). Although research in this area is limited due to the rarity of the disease, it is clear that the care of SMA patients poses a challenge for health care systems, insurance companies, and governments. Evaluating the long-term cost, benefits and burden of these interventions requires transparent data sharing among centers and health care organizations.

## The role for implementation science

The application of implementation science in the field of rare diseases, including neuromuscular disorders is limited, mainly due to the lack of successful therapies. Although each disease affects a small number of patients by definition, rare diseases collectively impact more than 350 million patients globally (38). With most of these diseases caused by monogenic mutations, it is very likely that many cell and gene therapies will receive approval in the near future. Application of implementation science in SMA could serve as a model for other rare conditions that will soon have disease specific treatments.

Core concepts in implementation science include a focus on evaluating multilevel contextual factors (e.g., policy, organizational, and individual factors) that influence the implementation process, the evaluation strategies for implementing evidence-based practices, and the application of frameworks to guide the full spectrum of translational research. In particular, determinant frameworks, such as the consolidated framework for implementation research (CFIR) provide an ideal platform for the understanding of barriers and enablers that affect implementation outcomes in SMA (39, 40). The identification of specific constructs related to the intervention, in this case the diagnosis and treatment of patients with SMA, the inner and outer settings, patient characteristics and processes allows for a systematic approach to these multidimensional matters. CFIR could be used to guide the continuous improvement of newborn screening programs, both at the institutional level and state level.

**Figure 1:**
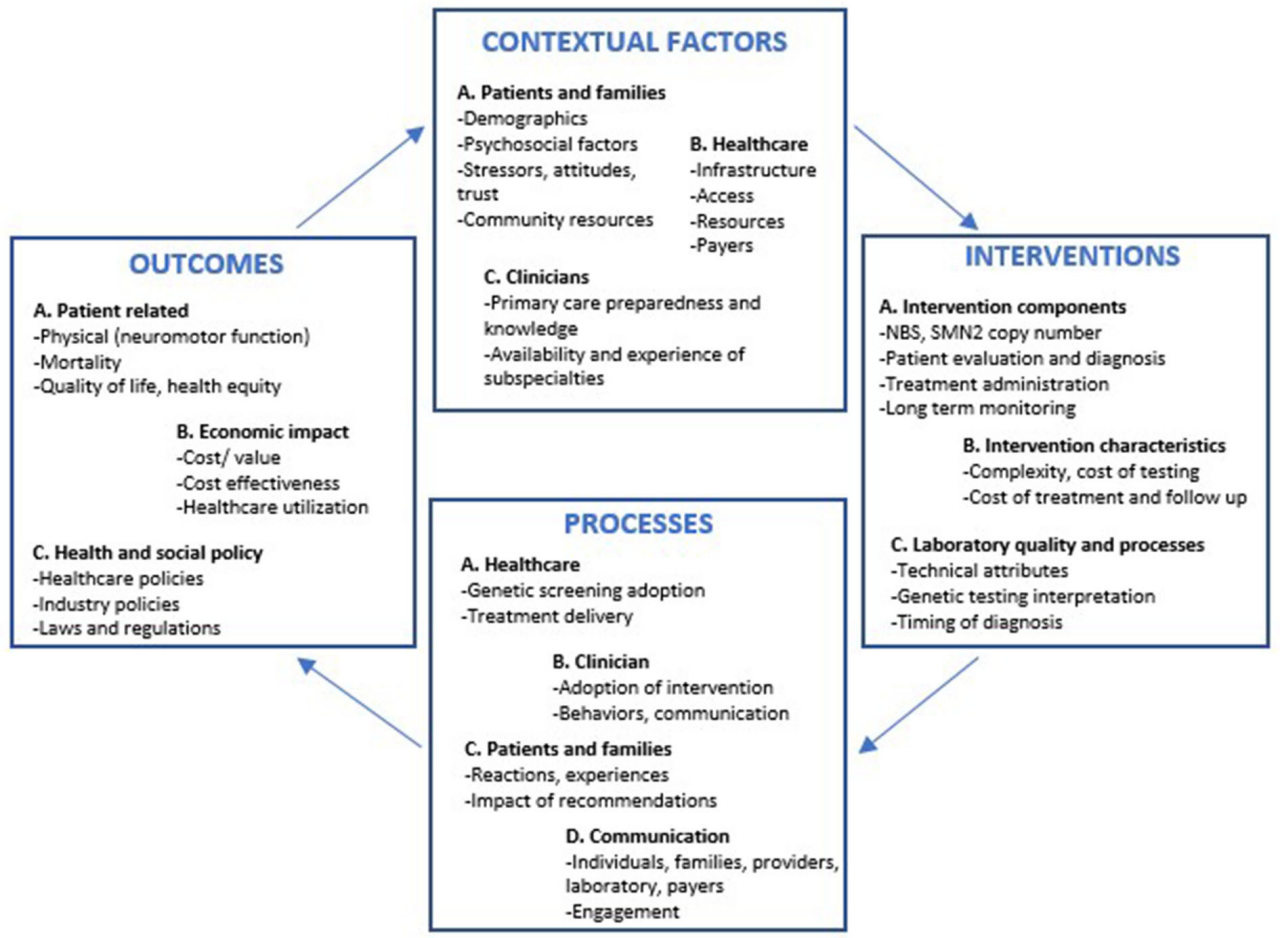
GMIR framework adapted for infants with spinal muscular atrophy diagnosed *via* newborn screening programs. NBS, Newborn screening; SMN2, Survival motor neuron 2 gene.

Informed by the CFIR and other implementation science frameworks, a comprehensive framework has recently been proposed to address the specific needs of precision medicine implementation efforts. Given the complexity of genomic medicine and the recognition that existing frameworks may lack sufficient flexibility to address the full spectrum of clinical and research needs, the Genomic Medicine and Integrative Research (GMIR) framework was developed by the clinical sequencing evidence generating research consortium (CSER) and the Implementing Genomics in Practice (IGNITE) network, both consortia funded by the NIH (41). This framework sheds light on priority research domains and factors across multiple levels, including affected individuals, providers, health systems and the social environment, a platform that can accommodate the challenges of precision medicine (41). Although to date no framework fits the specific needs of patients with SMA, an adaptation of GMIR outlining the steps for the implementation of interventions can be used to inform the care of children with SMA (Figure 1) (41). This framework highlights the contextual factors that facilitate early diagnosis and treatment. In addition, the GMIR provides a detailed overview of the different interconnected subdomains representing the processes and outcomes related to SMA, ensuring that all aspects related to treatment implementation and sustainability are continuously assessed, including those pertinent to the patient, their caregivers and the community, in response to changes in healthcare and industry policies.

For example, D'Silva et al. propose a model of care for the diagnosis and delivery of Zolgensma^®^ based on experience in Australia (42). Despite the lack of an overarching implementation science framework, specific implementation strategies are recommended to address the challenges that result from variations in diagnostic modalities, AAV9 testing, coordination of stakeholders, geographical location and workforce utilization (42).

While determinants frameworks are critical to guide the study of implementation barriers and facilitators, evaluation frameworks are necessary to measure implementation success. One such framework is the RE-AIM (reach, efficacy, adoption, implementation and maintenance) model, a widely applied framework to determine the overall impact of an intervention at the individual and population based level (43). RE-AIM can successfully serve as a tool for the evaluation of newborn screening programs and patient registries, as these interventions require the involvement of a range of organizations and personnel. If adequately performed, the application of RE-AIM and other evaluation frameworks can prove to be a powerful tool in the field of SMA and other rare diseases.

## Conclusions

Although the implementation of newborn screening and the opportunity to treat minimally symptomatic infants have, without a doubt, led to improvements in the devastating outcomes of patients with SMA, variation in the execution of the diagnostic and treatment processes pose remarkable challenges. Once a tragical disease, SMA is now a chronic condition with multiple phenotypes and evolving medical needs. With the common goal to provide optimal care for patients with SMA, diagnostic and treatment centers should work together along with patients and their families, clinicians, pharmaceutical companies and governmental agencies to ensure not only timely treatment and follow up, but also the development of the processes necessary for the evaluation of outcomes beyond efficacy and safety, such as cost-effectiveness and program sustainability. These and other strategies can play a key role in facilitating the development of effective and reproducible models for the care of children with rare diseases that will benefit from similar approaches in the upcoming years. Well planned implementation strategies will also allow stakeholders to learn about additional features such as the identification of new phenotypes, detection of unknown consequences of novel therapies, comorbidities and outcomes, that would otherwise be almost impossible to evaluate given the limited number of patients, while developing cost effective protocols for state-of-the-art care and detailed long-term surveillance.

## Data availability statement

The original contributions presented in the study are included in the article/supplementary material, further inquiries can be directed to the corresponding author.

## Author contributions

CL-A, BB, and RS contributed to the conception and design of the work. CL-A drafted the manuscript. BB and RS performed a critical review of the manuscript. All authors reviewed and approved the final version of the manuscript.
